# Type of Mask May Impact on Continuous Positive Airway Pressure Adherence in Apneic Patients

**DOI:** 10.1371/journal.pone.0064382

**Published:** 2013-05-15

**Authors:** Jean Christian Borel, Renaud Tamisier, Sonia Dias-Domingos, Marc Sapene, Francis Martin, Bruno Stach, Yves Grillet, Jean François Muir, Patrick Levy, Frederic Series, Jean-Louis Pepin, on behalf of the Scientific Council of The Sleep Registry of the French Federation of Pneumology (OSFP)

**Affiliations:** 1 HP2 Laboratory INSERM U 1042, University Grenoble Alpes, Grenoble, France; 2 Pôle Locomotion, Rééducation et Physiologie, Hôpital Albert Michallon, Grenoble, France; 3 Research and Development department, AGIRadom, Meylan, France; 4 Pneumology and Respiratory Intensive Care U, Rouen University Hospital, Rouen, France; 5 Institut Universitaire de Pneumologie et Cardiologie de Québec, Université Laval, Québec, Canada; 6 Unité Sommeil et Vigilance, Polyclinique Bordeaux Cauderan, Bordeaux, France; 7 Unité des pathologies du sommeil, Centre hospitalier de Compiègne, Compiègne, France; 8 Pneumologie, Cabinet Médical Saint Michel, Valenciennes, France; 9 Pneumologie, Cabinet Médical, Valence, France; 10 Scientific Council of The Sleep Registry of the French Federation of Pneumology, (FFP), Paris, France; Clinica Universidad de Navarra, Spain

## Abstract

**Rationale:**

In obstructive sleep apnea patients (OSA), continuous positive airway pressure (CPAP) adherence is crucial to improve symptoms and cardiometabolic outcomes. The choice of mask may influence CPAP adherence but this issue has never been addressed properly.

**Objective:**

To evaluate the impact of nasal pillows, nasal and oronasal masks on CPAP adherence in a cohort of OSA.

**Methods:**

Newly CPAP treated OSA participating in “Observatoire Sommeil de la Fédération de Pneumologie”, a French national prospective cohort, were included between March 2009 and December 2011. Anthropometric data, medical history, OSA severity, sleepiness, depressive status, treatment modalities (auto-CPAP versus fixed pressure, pressure level, interface type, use of humidifiers) and CPAP-related side effects were included in multivariate analysis to determine independent variables associated with CPAP adherence.

**Results:**

2311 OSA (age = 57(12) years, apnea+hypopnea index = 41(21)/h, 29% female) were included. Nasal masks, oronasal masks and nasal pillows were used by 62.4, 26.2 and 11.4% of the patients, respectively. In univariate analysis, oronasal masks and nasal pillows were associated with higher risk of CPAP non-adherence. CPAP non-adherence was also associated with younger age, female gender, mild OSA, gastroesophageal reflux, depression status, low effective pressure and CPAP-related side effects. In multivariate analysis, CPAP non-adherence was associated with the use of oronasal masks (OR = 2.0; 95%CI = 1.6; 2.5), depression, low effective pressure, and side effects.

**Conclusion:**

As oronasal masks negatively impact on CPAP adherence, a nasal mask should be preferred as the first option. Patients on oronasal masks should be carefully followed.

## Introduction

Continuous positive airway pressure (CPAP) is the first line therapy for moderate to severe obstructive sleep apnea syndrome (OSAS) [1]. Both randomized controlled studies and observational cohort studies have demonstrated beneficial effects in terms of cardiovascular [2], [3], [4], [5], metabolic [6], daytime vigilance and quality of life [7] outcomes. However, on a long term basis, 20 to 25% of OSAS patients discontinue CPAP treatment [8], [9] although CPAP adherence is crucial to improve symptoms [10] and cardiometabolic outcomes [2], [4], [11], [12] with a dose effect relationship [13], [14].

As a consequence, improving CPAP usage in poorly-adherent patients remains a major challenge for physicians and care givers. Many studies have been dedicated to delineate the factors associated with the risk of non-adherence. Factors of different natures are likely to influence CPAP tolerance and adherence: *i)* patients’ characteristics (age, psychological factors [15], [16], marital and employment status [8]), *ii)* OSAS severity [17] and related symptoms [18], [19] as well as *iii)* technical innovations concerning both the device itself or interfaces [20].

Clinicians generally consider that the choice of the interface is crucial, although insufficient, for the success of CPAP treatment. As a first option, nasal masks are the most frequently used interface [21], [22]. Oronasal masks, that cover both the nose and mouth, are proposed as a useful alternative in response to sleep-related mouth leaks (pressurized air escaping via the mouth when a nasal mask is used) [21], [23], [24] and nasal pillows have recently provided the opportunity to reduce mask size [25]. Two recently published case-reports suggest that in subgroups of patients, CPAP may not be effective when an oronasal mask or nasal pillows are used [26], [27]. Only very few studies have specifically looked at the impact of different masks types on CPAP adherence. Indeed, in a Cochrane analysis addressing the impact of delivery interfaces on CPAP adherence [22], only 132 patients from four randomized controlled trials were analyzed and no clear conclusions could be made: *“Due to the limited number of studies available comparing various interface types, the optimum form of CPAP delivery interface remains unclear »*. However, small sample size studies have suggested that the type of interface may impact on CPAP tolerance and adherence [25], [28]. The objective of this observational study was to evaluate, in a large prospective cohort of unselected OSAS patients, the impact of nasal pillows, and nasal and oronasal masks on CPAP adherence among other covariates (patients’ characteristics, sleep apnea severity, others technical aspects of CPAP treatment and side-effects), which are likely to influence CPAP adherence.

## Materials and Methods

### Study Design and Data Source

This is a prospective observational cohort study from the research database of the *«Observatoire Sommeil de la Fédération de Pneumologie”* (OSFP). This database is a large, well maintained database administered on a not-for-profit basis by the French Federation of Pneumology. It contains anonymized medical records from respiratory physicians in private practice, general hospitals and university hospitals [29]. The OSFP registry is a standardized web-based report including longitudinal data from patients complaining about sleep symptoms and being treated for sleep breathing disorders. Participating staff are trained in the use of computerized medical records and appropriate software. Periodic quality control checks are made to ensure up-to-standard data recording.

Ethical committee approval was obtained by “Le Comité consultatif sur le traitement de l’information en matière de recherche en santé” (C.C.T.I.R.S n° 09.521) and authorization from the “Commission Nationale Informatique et Liberté” (C.N.I.L), the French information technology and personal data protection authority. The OSFP Independent Scientific Advisory Committee approved data use for this study. Patients included in the database gave written informed consent.

#### Inclusion criteria

Between March 2009 and December 2011, adult patients (≥18 years old) fulfilling the following criteria were identified in the OSFP database:

- A baseline medical visit reported in the database that included the diagnosis of obstructive sleep apnea syndrome exclusively (i.e: patients with central or mixed apnea syndrome were not selected) and the prescription of CPAP treatment.
- A follow-up medical visit, within a 1 to 24 month window after CPAP prescription, reported in the database. Patients treated with other modalities of pressure therapy such as bi-level positive airway pressure were not selected.

#### Outcome measures

Clinical information collected for the analysis included anthropometry, medical history with cardiometabolic co-morbidities, severity of sleep apnea (established by in-lab attended or unattended polysomnography and respiratory polygraphy (minimum 3 cardiopulmonary channels)), daytime sleepiness (Epworth Sleepiness Scale; (ESS)) [30], fatigue (Pichot fatigue Scale), depressive status (Pichot depression scale [31]) and treatment modalities (auto-adjusted CPAP versus fixed pressure, pressure level, interface type, use of humidifier). A specific questionnaire targeting CPAP-related side effects was filled-in by each patient. All the data concerning treatment modalities were collected during the follow-up visit. Objective CPAP adherence, reported in hours/night, was obtained from built-in time counters on the CPAP devices over a period of at least one month preceding the follow-up visit. A threshold of four hours of CPAP usage per night was used to separate adherent and non-adherent patients [2], [4], [10], [11], [32].

### Exclusion Criteria

Patients were excluded from the analysis when *i)* CPAP adherence was not specified; *ii)* type of interface item was not reported; *iii)* Epworth sleepiness scale (ESS) and/or Pichot fatigue scale and/or Pichot depression scale were not completed. These criteria were applied according to the primary objective of the study and considering that psychological factors [15], [16] as well as daytime sleepiness [19] contribute to continuous positive airway pressure adherence.

### Data Management and Statistical Analysis

Data were analyzed using Statistical Analysis System (SAS®) software version 9.1.3 (SAS Institute, Cary, NC, USA). Continuous data were expressed as mean (SD), and categorical data as percentage. Univariate conditional logistic regression models were used to compare all the variables between CPAP adherent and non-adherent patients (threshold 4 h/night). The delay to the follow-up visit was the matching factor (conditional factor): four strata were defined: ]1–6 months], ]6–12 months], ]12–18 months] and ] 18–24 months]. When log-linearity of a continuous variable was not respected, the variable was converted to dichotomous data (> or<the median value).

Variables which were associated with the risk of being non-adherent to CPAP in univariate analysis (p<0.10) were included in a multivariable conditional logistic regression model (backward selection). Co-linearity between variables (defined as p<0.2 and r>0.4) was verified by Pearson’s or Spearman’s coefficient or Cramer’s V2. Missing values were mostly ≤1% of observations except for SpO2<90% (expressed in % of recording time) with 15% missing, and depressive status (3%). The missing values were replaced by the variable’s median for continuous data and for categorical data by the most frequent value [33].

Finally, a mixed model, adjusted for the mean length of CPAP treatment, was used to compare i) the daily adherence, ii) the mean CPAP pressure and iii) the CPAP-related side effects according to the interface modality (oronasal mask, nasal mask or nasal pillows) Only p-values <0.05 were considered statistically significant.

## Results

**Figure 1:**
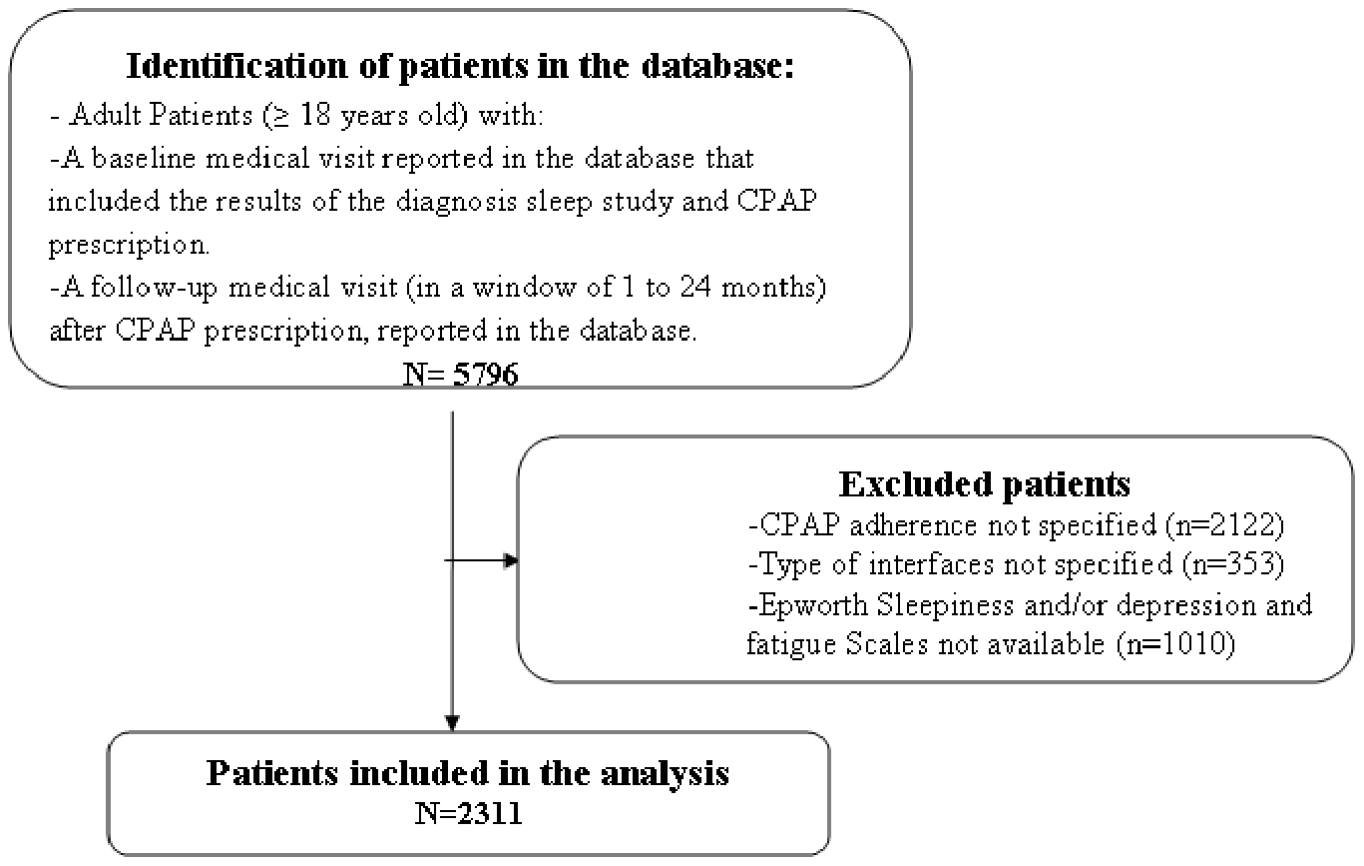
Flow Chart of the study.

Figure 1 shows the flow chart of the study. Two thousand three hundred and eleven OSAS patients with complete information regarding symptoms, comorbidities, CPAP technology, interfaces, CPAP adherence and side effects were analyzed. The mean delay between CPAP initiation and the follow-up visit was 4.5±3.6 months. Seventy-seven and nineteen percent of the follow-up visits occurred between 1 to 6 months and 6 to 12 months respectively.

For 2475 patients with OSA in the database, CPAP adherence was not known (2122 patients) or the type of interface was not recorded (353 patients) and they were not included in the analysis. Table 1 shows a comparison between patients who were analyzed versus patients excluded from the analysis. There were some statistically significant differences that actually had no clinical relevance.

**Table 1:** Comparison between patients included in the analysis and patients excluded because of missing adherence or type of mask data.

	Included	Excluded	P-value
	n = 2 311	n = 2 475	
**Anthropometrics**			
Age, Years	57.26±12.21	58.40±12.44	0.0016
BMI, kg/m^2^	32.16±6.58	31.80±6.85	0.0142
Gender, % female	29.0	26.5	0.0461
Current smokers, %	15.4	16.2	0.4728
**Sleep apnea characteristics**			
Apnea-hypopnea index, events/hour	40.65±20.55	40.53±20.96	0.9586
Depression, Fatigue and sleepiness scales			
Pichot Depression score	4.5±3.8	4.3±3.9	0.1004
Pichot Fatigue score	14.0±8.2	14.0±8.2	0.9033
Epworth sleepiness score	10.6±5.1	10.9±5.1	0.0476

### Patients’ Characteristics

Table 2 shows patients’ characteristics for the group as a whole. Seventy-one percent of the patients were male. The two main comorbidities encountered were hypertension and hypercholesterolemia. Eighty-seven percent of the patients were treated with auto-adjusted CPAP and 78.8% were adherent to CPAP (mean compliance ≥4 hours/night) at the follow-up visit.

**Table 2:** Patients’ characteristics (n = 2311).

**Anthropometrics**	
Age, *years*	57.26±12.21
BMI, *kg/m^2^*	32.16±6.58
Gender, % female	29.0
Active smokers, %	15.4
**Medical History**	
COPD, %	5.00
Hypertension, %	48.10
Myocardial infarction, %	4.00
Coronary artery disease, %	6.00
Heart failure, %	2.70
Arrhythmias, %	8.70
Stroke, %	3.00
Gastroesophageal reflux, %	20.90
Diabetes, %	17.20
Hypercholesterolemia, %	33.50
Hypertriglyceridemia, %	8.20
Depression, %	15.30
**Sleep apnea severity**	
Apnea-hypopnea index, events/hour	40.65±20.55
SpO2<90%, % of recording time	16.32±19.72
Epworth Sleepiness score	10.6±5.1
**Characteristics of CPAP treatment**	
CPAP use, hours/night	5.39±1.92
Patients with ≥4 hours/night, %	78.8
CPAP modality, % auto-adjusted pressure	86.90
Type of interface, %	
Nasal	62.40
Oronasal	26.20
Nasal Pillows	11.40
Additional heated Humidification (%)	31.40

### Factors Associated with Risk of CPAP Non-adherence in Univariate Analysis

**Figure 2:**
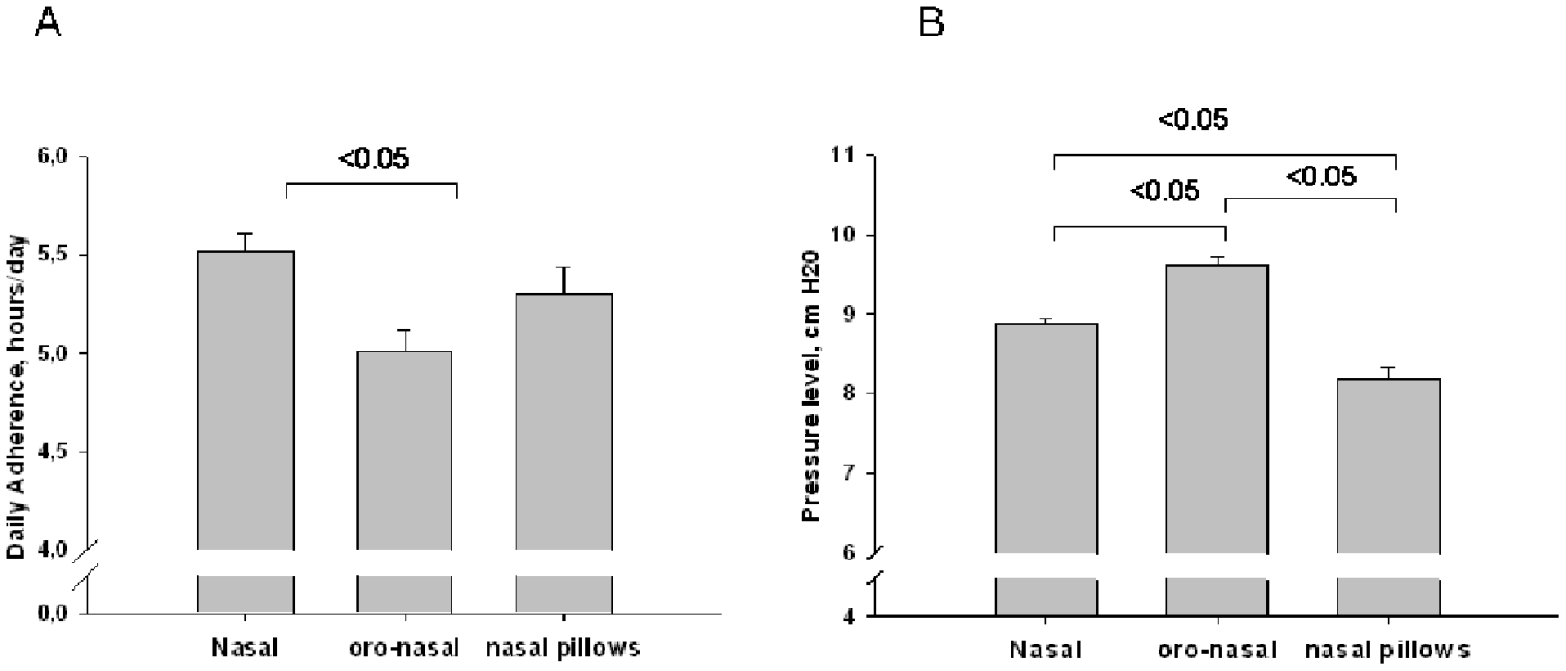
Adherence (a) and positive airway pressure level (b) according to the type of interface.

Tables 3 and 4 display the variables according to CPAP adherence status. Factors associated with the risk of being non-adherent to CPAP can be arbitrarily separated into three domains: i) general patient characteristics (Table 3), ii) sleep apnea severity (Table 4), iii) technical aspects of CPAP treatment, delivery interfaces and side-effects (Table 4). Regarding the technical aspects of CPAP treatment, and particularly the types of interface, both oronasal masks and nasal-pillows were associated with a higher risk of non-adherence compared to a nasal mask (Figure 2a).

**Table 3:** General patient characteristics according to CPAP adherence status and risks of being non-adherent (univariate analysis).

	CPAP adherence <4 h/night	CPAP adherence ≥4 h/night	Odds Ratio	95% CI	P-value
	n = 491	n = 1820			
**Antropometrics**					
Age, Years	56.07±12.31	57.58±12.17	0.990	[0.982; 0.998]	0.0194
BMI (≥31.10 vs <31.10), kg/m^2^	31.76±6.34	32.27±6.64	1.011	[0.828; 1.234]	0.7202
Gender, % female (male vs female)	32.8	28.0	0.802	[0.647; 0.994]	0.0442
Current smokers, %	18.10	14.70	1.281	[0.984; 1.668]	0.0658
**Medical History**					
COPD, %	6.30	4.60	1.414	[0.924; 2.162]	0.1103
Hypertension, %	46.40	48.50	0.928	[0.760; 1.134]	0.4656
Myocardial infarction, %	3.70	4.10	0.925	[0.547; 1.566]	0.7720
Coronary artery disease, %	6.50	5.90	1.140	[0.757; 1.716]	0.5313
Heart failure, %	2.90	2.70	1.073	[0.587; 1.961]	0.8185
Arrhythmias, %	7.10	9.10	0.772	[0.528; 1.127]	0.1801
Stroke, %	3.50	2.90	1.214	[0.696; 2.119]	0.4947
Gastroesophageal reflux, %	24.80	19.90	1.345	[1.063; 1.702]	0.0135
Diabetes, %	18.30	16.90	1.102	[0.850; 1.429]	0.7022
Hypercholesterolemia, %	32.20	33.90	0.930	[0.751; 1.1150]	0.5018
Hypertriglyceridemia, %	9.00	8.00	1.168	[0.820; 1.663]	0.3908
Upper airway surgery for snoring, %	2.04	2.16	0.914	[0.454; 1.841]	p = 0.8012
**Depression, Fatigue and sleepiness scales**					
Pichot Depression score	5.08±4.00	4.33±3.78	1.051	[1.025; 1.078]	0.0001
Pichot Fatigue score (≥14.00 vs <14.00)	14.93±8.51	13.79±8.17	1.398	[1.143; 1.711]	0.0011
Epworth sleepiness score (≥or <10)	10.85±5.07	10.54±5.10	1.191	[0.972; 1.459]	0.0917

**Table 4:** Sleep breathing disorders severity, technical aspects of CPAP treatment and side-effects according to CPAP adherence status and risks of being non-adherent (univariate analysis).

	CPAP adherence <4 h/night	CPAP adherence ≥4 h/night	Odds Ratio	95% CI	P-value
	n = 491	n = 1820			
**Sleep apnea characteristics**					
Apnea-hypopnea index, events/hour	37.76±19.61	41.43±20.73	0.991	[0.986; 0.996]	0.0007
SpO2<90%, % of recording time	14.07±18.42	16.91±20.02	0.992	[0.986; 0.998]	0.0143
**Technical aspects of CPAP treatment**					
CPAP treatment modalities					
auto-adjusted pressure vs constant, %	88.20	86.50	1.160	[0.854; 1.574]	0.3415
Additional heated Humidification, %	31.40	31.40	0.989	[0.798; 1.227]	0.9219
Type of interface, % (nasal as reference)					
Nasal	50.30	65.70	Overall test		<0.0001
Oronasal	37.30	23.20	2.090	[1.676; 2.608]	<0.0001
Nasal pillows	12.40	11.10	1.439	[1.047; 1.978]	0.0249
Effective pressure level (≥9 vs <9 cmH2O)	8.64±2.44	9.09±2.38	0.768	[0.623; 0.946]	0.0013
**CPAP-related side-effects**					
Nasal Congestion, %	15.90	11.20	1.485	[1.119; 1.970]	0.0061
Ocular irritation, %	8.10	6.40	1.301	[0.895; 1.892]	0.1677
Dry mouth, %	33.40	23.20	1.650	[1.328; 2.050]	<0.0001
Choking sensation under CPAP, %	23.60	6.30	4.603	[3.472; 6.102]	<0.0001
Headaches, %	3.50	3.10	1.115	[0.643; 1.935]	0.6984
Psychologically percieved inconveniance, %	28.50	10.50	3.403	[2.658; 4.357]	<0.0001
Family tolerance, %	7.30	7.40	1.000	[0.682; 1.466]	0.9993
Aerophagia, %	4.30	3.80	1.116	[0.678; 1.837]	0.6669

**Figure 3:**
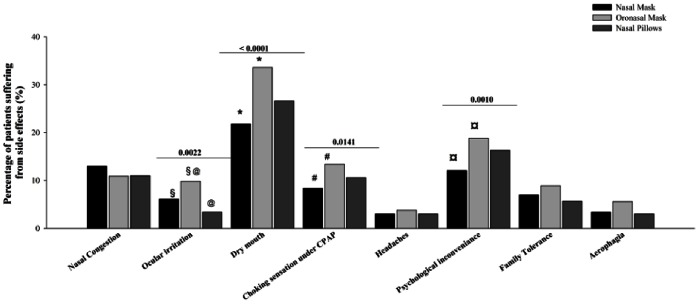
Percentage of side effects according to the type of mask. -***for ocular irritation*** § = difference between nasal mask and oronasal mask with p = 0.049; @ = difference between oronasal mask and nasal pillows with p = 0.062. ***-for dry mouth***: * = difference between nasal mask and oronasal mask with p<0.0001. ***-for Choking sensation under CPAP***: # = difference between nasal mask and oronasal mask with p = 0.0024. - ***for psychologically perceived inconvenience***: ¤ = difference between nasal mask and oronasal mask with p = 0.0001.

Interestingly, the lower the effective pressure level, the higher the risk of non-adherence. Oronasal masks were associated with higher pressure levels than both nasal masks and nasal-pillows (Figure 2b) (mixed model analysis). Finally, among CPAP-related side-effects, nasal congestion, dry mouth, choking sensation under CPAP and psychologically perceived inconvenience, were associated with a higher risk of non-adherence. As shown in Figure 3, the proportion of patients reporting side-effects was significantly larger with oronasal masks than with nasal masks (using a mixed model, adjusted for the mean length of CPAP treatment).

Regarding general patient characteristics (Table 3) and sleep apnea severity (Table 4), the risk of being non-adherent to CPAP was increased for females and younger patients. Similarly, the higher scores of depression and fatigue were associated with a greater risk of non-adherence. The presence of gastroesophageal reflux also increased the risk of non-adherence. In contrast, most severe sleep apnea syndromes, objectively recognized by the Apnea-hypopnea Index and time spent with a nocturnal SpO2<90%, were associated with a lower risk of non-adherence to CPAP.

### Factors Associated with Risk of CPAP Non-adherence in Multivariate Analysis

**Figure 4:**
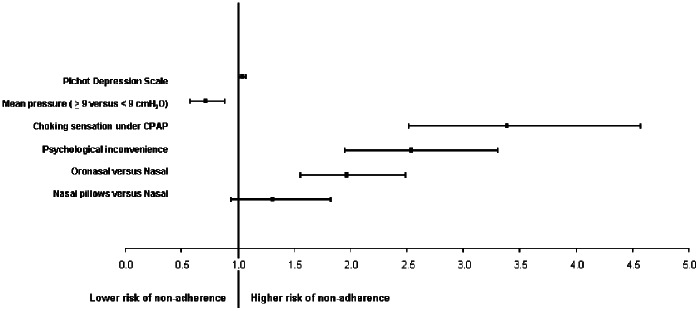
Variables associated with the risk of being non-adherent to CPAP in a multivariable conditional logistic regression model.

The following variables were included in the multivariate analysis: age, Pichot fatigue and depression scores, SpO2<90% (% of recording time), effective pressure level, gender, gastroesophageal reflux, type of interface, nasal congestion, dry mouth, choking sensation under CPAP, psychologically perceived inconvenience. Although the Pichot fatigue score and depression score were collinear variables, both were included in the multivariate model in view of their respective clinical relevance. Nasal congestion and a choking sensation under CPAP were also both included in the model for the same reason (clinical relevance). Figure 4 displays variables associated with the risk of being non-adherent to CPAP in a multivariable conditional logistic regression model.

Regarding the technical aspects of CPAP treatment and CPAP-related side effects, the type of interface (oronasal mask compared to nasal mask), a low effective pressure level, choking sensation under CPAP and psychologically perceived inconvenience were associated with a higher risk of non-adherence. Among general patient characteristics, depressive status remained the only independent factor associated with an increased risk of non-adherence.

## Discussion

The main objective of this prospective cohort study was to evaluate the impact of different interfaces on CPAP adherence among other potentially confounding variables. The results can be summarized as follows: (i) In univariate analysis, multiple factors were identified as linked to CPAP non-adherence (ii) in multivariate analysis, the type of interface, depressive status, a low effective pressure, a choking sensation when wearing CPAP and psychologically perceived inconvenience related to CPAP treatment were independently associated with a higher risk of CPAP non-adherence. (iii) The oronasal mask was associated with a lower adherence than nasal masks and required higher pressure levels than both nasal masks and nasal-pillows.

Methodological considerations related to the study design should to be discussed: Firstly, in a national registry such as this, we cannot guarantee that the same care and medical attention were provided in all participating centres. This is a common concern for many prospective observational cohorts [2], [3]. For this reason, large sample sizes are required to diminish the impact of such limitations. Secondly, there were some statistically significant differences between patients included in the analysis versus patients excluded. Although these differences are not likely to be clinically relevant, this limits the generalizability of our findings.

Our study is the first to suggest an influence of the type of mask on CPAP adherence in a large prospective unselected cohort of OSA patients. This is corroborated by unpublished data from the observatory of “Association Nationale pour le Traitement à Domicile de l’Insuffisance Respiratoire” (ANTADIR, http://www.antadir.com/), a federation of non-profit French regional associations delivering home CPAP treatment [34], which has recently collected data from 5892 OSA patients treated with CPAP for 5 to 12 months and has found that CPAP adherence was significantly lower in patients using facial masks (oronasal) than patients using nasal masks (5.1(2.3) versus 5.7(2.2) hour/night p<0.0001). These data however were not controlled for confounders. Previously, only one small (n = 20) randomized control study [28] has suggested that patients using an oronasal mask exhibited a lower adherence than those equipped with a nasal mask after 1-month of follow-up. In contrast, the three other randomized control studies, performed with a short-term follow-up (3 to 8 weeks) in CPAP-naïve patients, failed to demonstrate any significant impact of type of mask on CPAP adherence [25], [35], [36]. Two of these four studies [35], [36] compared a nasal mask versus an intraoral mask (this latter type of mask was not represented in our study), and these studies suggested that the type of interface did not influence CPAP adherence in highly selected patients. In our prospective cohort of unselected patients, corresponding to real life, nasal masks are often the first line of interface used and other types of masks are principally considered to counteract adverse effects such as mouth leaks or nasal intolerance [22]. Thus, the significant influence of the type of mask on CPAP adherence found in the present study may be partly explained by the fact that the reasons to start with nasal, oronasal or pillows and the history of different masks used by the patient before the follow-up visit were not available. It could be argued that oronasal masks were more frequently used as second intention masks reflecting a difficult initiation to CPAP treatment. However, the present results show that the proportion of side-effects was significantly larger with oronasal masks than with nasal masks. As a consequence, our results demonstrate that if an oronasal mask was only proposed in second intention to problematic patients (with poor adherence to nasal CPAP and/or with side effects), then, the oronasal mask neither adequately resolved the problem of adherence nor the problem of side effects. Mouth dryness owing to mouth leaks is one of the more frequent side effects reported by the patients. These mouth-leaks are also inter-related with nasal congestion and have been related to poor compliance [24]. Indeed, Bachour et al [23] have compared two groups of apneic patients, free of nasal symptoms, during an attended polysomnography without CPAP. One group of patients spent more than 70% of their total sleep time in mouth breathing (considered as “*mouth breathers*”) and the other group spent less than 30% in mouth breathing *(“nasal breathers”)*. “*Mouth breathers”* when treated exhibited a lower adherence to nasal-CPAP than *“nasal breathers”* after three months and one year of follow up. Oronasal masks have been designed to overcome this problem of mouth leaks and therefore potentially improve CPAP adherence. Nevertheless, because of greater difficulties in fitting [37] or higher probability of displacement during sleep, several recent studies have shown that non-intentional leaks remained more frequent with oronasal mask than with nasal masks [37], [38], [39]. Regardless of the type of interface, these leaks have been shown as being associated with a higher risk of non-adherence [40]. Furthermore, we have recently demonstrated that wearing oronasal masks is associated with an increase in upper airway resistance [41] due to the posterior displacement of the tongue and may lead to higher residual respiratory events than nasal masks [37], [38]. This may contribute to a lack of clinical benefit and as a consequence further reduce CPAP adherence. In line with the increase in upper airway resistance induced by oronasal masks, we demonstrated that oronasal masks were associated with higher therapeutic pressure than nasal masks in the present study. This is in accordance with Ebben et al. [39]. We could speculate that a higher CPAP pressure is required to counteract the posterior displacement of the mandibular induced by an oronasal mask (i.e constraint on the chin and traction of straps) and high pressure levels may in turn increase the risk of unintentional leaks. It is important to note that our results show that a low effective pressure level was associated with a higher risk of non-adherence. This is in accordance with Kohler et al [17] who have previously shown that higher CPAP pressure and greater apnea severity were associated with a higher probability to pursue CPAP treatment (in univariate analysis). Similarly, Valentin et al [40] also found that non-adherent patients were being treated with lower pressure than adherent patients. Taken together, these findings imply that the side-effects rather than the effective pressure level itself contribute to CPAP non-adherence.

The type of interface was not the single factor that influenced CPAP adherence. Depressive status and psychologically perceived inconvenience contributed to modulate this adherence. Gagnadoux et al [8], in a prospective cohort, failed to demonstrated a link between depressive status and adherence. Furthermore, Poulet et al [15] showed that adherent patients tended to have a worse depression score. This contrast between our results and these previous studies highlights the need of large scale studies focusing on the influence of psychological factors. Moreover, it also underscores the need to pursue interventions that could help patients to overcome barriers such as psychologically perceived inconvenience and to improve active coping processes. Educational programs are important but difficult to implement and to achieve, and need experienced care givers. Finally, in line with a previous study [8], [17], our study failed to demonstrate that sleepiness was associated with CPAP adherence. One could argue that the mean Epworth score was low and thus represented a selection bias. However, this score was in accordance with the mean ESS value previously reported in the ESADA cohort [42].

Our study is important as it proposes a very simple and probably important clinical message; that the use of oronasal masks should be restricted to cases of documented nasal mask failure. In term of costs, it should also be noted that the price of an oronasal mask is two to three-fold the price of a nasal mask and proper indications may lead to significant cost reductions. Better definition of the appropriate indications for each type of interface would also lead to substantial reductions in CPAP-related costs.

### Conclusions

The present study is the first to suggest an influence of the type of mask on CPAP adherence in a large cohort of OSA patients. Compared to nasal masks, oronasal masks increased the risk of being non-adherent. A large scale randomized controlled study is required to confirm these results. However, as oronasal masks may negatively impact on CPAP adherence, a nasal mask should be preferred as the first option when initiating CPAP treatment. Patients on oronasal masks should be carefully followed. Finally, new strategies such as the combination of a nasal mask and mandibular advancement device [41], [43] should be tested in a randomized controlled trial in patients presenting excessive mouth leaks with nasal CPAP.
